# Changes in Parental Reflective Functioning before and after a postpartum depression group therapy

**DOI:** 10.1192/j.eurpsy.2024.313

**Published:** 2024-08-27

**Authors:** L. Erkoreka, Z. Alonso, L. Pérez Cabeza

**Affiliations:** ^1^Galdakao-Usansolo University Hospital, Galdakao; ^2^University of the Basque Country UPV/EHU, Leioa; ^3^Biocruces Bizkaia Health Research Institute, Barakaldo; ^4^Bizkaia Mental Health Network, Getxo; ^5^Bizkaia Mental Health Network, Bilbao, Spain

## Abstract

**Introduction:**

Parental Reflective Functioning (PRF) refers to parents’ ability to view their children’s and their own behavior by considering internal mental states, such as thoughts, desires, and intentions. Depression has been described as compromising reflective functioning in female samples, whereas other studies have not detected differences in RF between depressed and non-depressed mothers.

**Objectives:**

We aim to study whether a group intervention focused on postpartum depression, which we have already observed to cause significant changes in the mother-child bond and the severity of depressive, also improves parental reflective functioning.

**Methods:**

To that end, we analyzed pre-post data from two different groups (N=12), composed of mothers who had been clinically diagnosed with postpartum depression. They received the 6-week Mothers & Babies Program© and completed the Parental Bonding Questionnaire (PBQ), the Edinburgh Postnatal Depression Scale (EPDS) and the Parental Reflective Functioning Questionnaire (PRFQ) before and after group therapy. Pre-post data from the PRFQ were analyzed using the repeated measures t-test. The correlation between changes in the three questionnaires was also analyzed using Pearson’s correlation test.

**Results:**

Significant changes were observed in the Pre-Mentalization Modes (pre=2.37±.457, post=2.03±.520, t=2.0206, p=0.027) and Certainty About Mental States (pre=2.87±1.141, post=3.68±.908, t=-2.814, p=0.010) subscales of the PRFQ, with no significant changes in the Interest and Curiosity subscale (t=-.516, p=0.309). A significant correlation was also observed between pre-post change in EPDS scores and pre-post change in the Certainty About Mental States subscale of the PRFQ (r=-.640, p<.05), while no significant correlations were observed with the rest of the PRFQ subscales, nor with the PBQ.

**Conclusions:**

A brief cognitive-behavioral group therapy developed specifically to treat postpartum depression improves pre-post scores on the Pre-Mentalization (lower post- than pre- score) and Certainty About Mental States (higher post- than pre- score) subscales of the PRFQ. Although a control group is needed to determine the actual effect of the intervention, as time could also play a role in the observed changes, this is an encouraging result. Moreover, the improvement obtained in Certainty About Mental States is inversely correlated with the pre-post changes observed in the EPDS, meaning that the greater the improvement in depression, the greater the improvement in the aforementioned subscale of the PRFQ. A larger sample is needed to assess a hypothetical mediating effect of depression in the observed change.

**Disclosure of Interest:**

None Declared

